# Instagram addiction, life satisfaction and self esteem in young adults

**DOI:** 10.1192/j.eurpsy.2024.284

**Published:** 2024-08-27

**Authors:** G. Reis, B. R. Maia

**Affiliations:** ^1^Universidade Católica Portuguesa; ^2^Universidade Católica Portuguesa, Center for Philosophical and Humanistic Studies, Braga, Portugal

## Abstract

**Introduction:**

Addiction to Instagram can have severe consequences at a psychological, physiological and social level. On the other hand, social networks can be useful tools for an individual’s daily life. Studies show that the problematic use of some social networks, namely Instagram, can have an impact on users’ self-esteem. This construct is considered a predictor of life satisfaction, which is why in the literature these two variables appear positively related.

**Objectives:**

To explore the relationship between addiction to Instagram, life satisfaction and self-esteem in young adult university students and to carry out a comparison between groups with and without probable addiction to Instagram.

**Methods:**

The sample was composed by 241 Portuguese university students with a mean age of 22.03, (SD = 2.29, range 18-29), and who have an Instagram account. Subjects fulfilled a sociodemographic questionnaire, and the Portuguese version of the Bergen Instagram Addiction Scale, the Life Satisfaction Scale and the Rosenberg Self-Esteem Scale.

**Results:**

The average score on the Bergen Instagram Addiction Scale was of 13.37 (*SD* = 4.41), with 29.5% of the sample spending one to two hours a day (*n* = 71) on the social network and 29.1% showing a probable Instagram addiction (*n* = 70). Mean scores of 27.17 (*SD* = 5.34) were found on the Rosenberg Self-Esteem Scale and 16.31 (*SD* = 3.97) on the Satisfaction with Life Scale. A strong relationship was found between life satisfaction and self-esteem, with males tending to have an higher self-esteem comparing to females. A low negative correlation was found between self-esteem and the total score on the Bergen Instagram Addiction Scale, which was not maintained when analyzing groups with and without a probable Instagram addiction.

**Conclusions:**

This study demonstrates the probable presence of instagram addiction and the potential role of low self-esteem. It also emphasizes the strong relationship between life satisfaction and self-esteem. Instagram updates, as well as habits developed during the pandemic period, may have worsened the instagram use. The study shows how paradoxical the impacts of using this social network can be. Furthermore, the present study raises awareness to new national investigations that explore the use of Instagram and how they are related to the impacts on users’ lives.

**Disclosure of Interest:**

None Declared

